# Mechanisms of salvianolic acids in kidney disease therapy: advances and perspectives

**DOI:** 10.3389/fphar.2026.1831031

**Published:** 2026-05-15

**Authors:** Jing Li, Xiaoqin Liu, Haoyue Feng

**Affiliations:** 1 Nephrology Depart. 1, Hospital of Chengdu University of Traditional Chinese Medicine, Chengdu, Sichuan, China; 2 Chengdu University of Traditional Chinese Medicine, Chengdu, Sichuan, China

**Keywords:** kidney diseases, pharmacological mechanisms, salvianolic acid A, salvianolic acid B, salvianolic acid C

## Abstract

Kidney diseases, including acute kidney injury (AKI), chronic kidney disease (CKD), nephrotic syndrome (NS) and diabetic nephropathy (DN), represent a major global public health concern. Salvianolic acids are water-soluble bioactive components from *Salvia miltiorrhiza*, among which salvianolic acid A (SAA), salvianolic acid B (SAB) and salvianolic acid C (SAC) are the focus of current research. This review systematically elaborates their renoprotective value, establishes a clear pharmacokinetic-pharmacodynamic (PK-PD) correlation, summarizes multi-target protective effects in multiple kidney disease models, and conducts in-depth horizontal comparisons of efficacy intensity, target affinity and pathway specificity among the three components, key pharmacokinetic parameters including half-life (T_1_/_2_), time to peak concentration (T_max_), peak plasma concentration (C_max_), and area under the curve (AUC). Low oral bioavailability, rapid metabolism and insufficient renal distribution are common bottlenecks that restrict therapeutic efficacy, especially long-term anti-fibrotic activity and chronic intervention effects. Accordingly, promising strategies such as kidney-targeted nanodelivery systems and structural modification are proposed, and the mechanism of enhancing efficacy by optimizing pharmacokinetic properties is clarified. This review improves the mechanistic understanding of salvianolic acids, strengthens the horizontal comparison of three components, and supports their further translation into clinical therapies.

## 1 Introduction

Kidney diseases, with their high incidence, disability, and mortality rates, have emerged as a major global public health challenge (Peek and Wilson, 2023; Petr and Thurman, 2023). These include acute kidney injury (AKI), chronic kidney disease (CKD), nephrotic syndrome (NS), diabetic nephropathy (DN), and lupus nephritis (LN) caused by systemic lupus erythematosus (SLE) affecting the kidneys (Fiaccadori et al., 2021; Lameire et al., 2021; Wang et al., 2023b). Although current treatment regimens play a role in controlling disease progression, they are often limited by suboptimal efficacy and significant side effects (Wang et al., 2023b). For example, a considerable proportion of CKD patients eventually progress to end-stage renal disease (ESRD), relying on dialysis or kidney transplantation to sustain life. This not only severely impacts patients’ quality of life but also imposes a heavy burden on healthcare resources (Wang et al., 2023b). Therefore, there is an urgent need to explore novel therapeutic strategies that can target multiple core disease mechanisms, are derived from natural sources, and offer a higher safety profile-a direction at the forefront of nephrology research.

Polyphenolic compounds, as one of the most widely distributed secondary metabolites in nature, have attracted sustained and in-depth scientific interest due to their remarkable biological activities and potential value in disease prevention and treatment. Polyphenols represent a large family, primarily including flavonoids, stilbenes, and phenolic acids, which are abundantly present in fruits, vegetables, tea, and numerous medicinal plants (Qin et al., 2019). *Salvia miltiorrhiza*, a renowned medicinal herb documented in the *Shennong Bencao Jing*, holds significant economic and therapeutic value and is widely used in Traditional Chinese clinical practice (Zhao et al., 2024). The active constituents of *S. miltiorrhiza* are mainly divided into lipophilic tanshinone and water-soluble salvianolic acids. Among these, the salvianolic acids serve as the key water-soluble components responsible for the herb’s pharmacological effects, with salvianolic acid A (SAA), salvianolic acid B (SAB), and salvianolic acid C (SAC) being the most abundant and biologically prominent representatives. Studies have reported that SAA, SAB, and SAC exhibit diverse biological activities, including anti-inflammatory, analgesic, antitumor, anticancer, and antioxidant properties (Fu et al., 2024; Li et al., 2025; Yue et al., 2025).

However, the three components show obvious differences in target affinity, efficacy intensity and disease adaptation due to slight structural variations. SAA is characterized by protecting peritubular capillaries and improving renal hypoxia; SAB has strong anti-fibrotic effects and clear direct targets; while SAC has specific pathway dependence but relatively weak efficacy and insufficient research evidence. Therefore, this article aims to organize and summarize the latest research progress on SAA, SAB, and SAC in various types of kidney diseases, including but not limited to AKI, CKD, diabetic nephropathy, nephrotic syndrome, and lupus nephritis. It delves into their individual and collective mechanisms, horizontal comparative characteristics of pharmacological activity, target affinity and evidence level, correlates pharmacokinetic profiles with pharmacological effects, and evaluates their potential for clinical translation and the associated challenges.

This review is intended to provide a systematic and comparative perspective on the nephroprotective effects of these natural compounds, clarify similarities and differences among SAA, SAB and SAC, and offer a solid theoretical foundation and novel insights for the future development of innovative drugs based on salvianolic acids, and for the integrated prevention and treatment of kidney diseases combining Traditional Chinese and Western medicine.

## 2 Safety evaluation and pharmacokinetics of salvianolic acids

### 2.1 Safety evaluation and pharmacokinetics of SAA

Preclinical and preliminary clinical studies on SAA have provided initial data regarding its safety profile. In a randomized, double-blind, placebo-controlled study involving 116 healthy Chinese subjects, single doses ranging from 10 to 300 mg and multiple doses of 60–200 mg were administered (Chen et al., 2022b). The results indicated that SAA was well-tolerated across all dose levels, with a generally low incidence of treatment-emergent adverse events (TEAEs) that did not appear to be dose-dependent. Pharmacokinetic analysis revealed that the main parameters after single-dose administration exhibited non-dose-proportionality within the tested dose range (Chen et al., 2022b). In animal models, toxicological studies following oral or intravenous administration of SAA have provided additional reference for safety evaluation (Yang H. et al., 2024). Acute toxicity studies indicated an LD50 of 1,161.2 mg/kg in mice after intravenous administration, while the lethal dose in beagle dogs ranged between 455 and 682 mg/kg (Yang et al., 2022). A 4-week subchronic toxicity study in beagles showed no adverse effects at an intravenous dose of 20 mg/kg. However, higher doses (80, and 300 mg/kg) induced transient effects on liver and kidney function and reversible thymus weight loss, suggesting a limited safety dose window (Yang et al., 2022).

Regarding absorption and bioavailability, a study involving oral administration in rats demonstrated a linear relationship between the maximum plasma concentration (C_max_) and dose, C_max_ was 31.53 μg/L at 5 mg/kg, 57.39 μg/L at 10 mg/kg, and 111.91 μg/L at 20 mg/kg. However, SAA exhibited low membrane permeability (apparent permeability coefficient less than 10^−6^ cm/s) in monolayers of human colorectal adenocarcinoma cells, which likely contributes to its limited oral bioavailability (Sun et al., 2018). Further studies in beagle dogs confirmed that SAA is rapidly absorbed after oral administration, reaching peak concentration within approximately 2 h, yet its absolute bioavailability remains low at only 1.47%–1.84%. Following intravenous administration in rats, the mean residence time (MRT) was about 2.91 h, and the half-life (T_1/2_) was approximately 1.96 h, indicating relatively rapid clearance *in vivo* (Sun et al., 2018; Sun et al., 2013). The rapid clearance and low bioavailability of SAA directly lead to insufficient renal tissue exposure, which significantly weakens its long-term protective effect on chronic kidney injury and continuous intervention efficacy. This pharmacokinetic defect explains why high doses or frequent administration are required in chronic models, and also supports the necessity of developing targeted delivery systems to improve renal distribution and prolong local action time.

### 2.2 Safety evaluation and pharmacokinetics of SAB

A substantial body of research indicates that SAB exhibits very low cytotoxicity *in vitro* and *in vivo* (Chanda et al., 2025). Safety assessments showed no significant pathological changes in the histoarchitecture of major organs, including the brain, heart, kidney, liver, and lungs (as observed by HE staining) following SAB treatment *in vivo* (Zhao et al., 2015). Similarly, Zhang et al. demonstrated that SAB could alleviate arsenic trioxide-induced cardiotoxicity *in vitro* and *in vivo* (Zhang et al., 2017). Another study reported that SAB exerts otoprotective effects in a transgenic zebrafish model, mitigating damage caused by ototoxic drugs through inhibition of the mitochondrial apoptotic pathway (Zheng et al., 2020). However, current research on the safety of SAB in humans remains limited. Among the few human studies, Liu et al. reported no significant side effects or toxic reactions in subjects receiving SAB. The study listed several adverse events considered possibly or potentially related to SAB, including vomiting (possibly related), common cold (suspected related), ventricular premature beats (suspected related), elevated fasting blood glucose (suspected related), and increased white blood cell count (suspected related). None of these events was accompanied by other abnormal symptoms (Liu et al., 2002). Based on available evidence, SAB appears to demonstrate favorable safety profiles in both animals and humans, with a low incidence of side effects and adverse events. Nevertheless, more systematic safety studies are still required to comprehensively assess its potential risks and to clarify its possible adverse reaction profile and underlying mechanisms.

The oral bioavailability of SAB is limited by multiple factors, including extraction methods, instability in the intestinal environment, poor membrane permeability, and susceptibility to structural degradation, which collectively result in restricted systemic exposure (Chanda et al., 2025; Shao et al., 2025). Following oral administration of 100 mg/kg in rats, the mean area under the concentration-time curve (AUC) was only 1.26 ± 0.36 μg·min/mL, with an absolute bioavailability as low as 0.022%. Notably, approximately 65% of the administered drug remained in the gastrointestinal mucosa even 4 h after dosing (Zhang et al., 2004). Comparative studies further revealed that the oral bioavailability of SAB in rats (3.90%) is lower than that of SAA (11.09%), underscoring its pronounced intestinal absorption barrier (Zhou et al., 2009). Regarding disposition characteristics in animals, intravenous administration of SAB magnesium (3, 6, and 12 mg/kg) in beagle dogs exhibited rapid distribution and elimination, the distribution half-life (T_1/2α_) ranged from 2.2 to 2.9 min, while the elimination half-life (T_1/2β_) was approximately 42–43 min (Li et al., 2004). The extremely short elimination half-life of SAB is a critical pharmacokinetic defect that directly limits its continuous inhibition of the TGF-β/Smad pathway and long-term anti-fibrotic activity. Since renal fibrosis is a chronic progressive process, the short *in vivo* retention time of SAB makes it difficult to achieve sustained target inhibition, which is an important reason for the discrepancy between *in vitro* potent anti-fibrotic activity and *in vivo* limited efficacy. It is noteworthy that at the highest dose (12 mg/kg), clearance and volume of distribution were lower compared to the lower dose groups, suggesting potential saturation of distribution or metabolism at elevated doses (Li et al., 2004). Furthermore, after intravenous injection of 100 mg/kg in rats, SAB concentration in bile rose rapidly and peaked within 30 min, consistently exceeding plasma levels, indicating significant biliary excretion likely mediated by active hepatic transport mechanisms (Chen et al., 2005). Studies in rabbit models similarly reported a low absolute oral bioavailability of approximately 5.6% (Ma and Wang, 2007).

### 2.3 Safety evaluation and pharmacokinetics of SAC

The oral absorption efficiency of SAC is low, with an absolute bioavailability in rats of only 0.29% ± 0.05%, indicating very poor gastrointestinal absorption (Song et al., 2016). Pharmacokinetic parameters in normal rats show an terminal elimination half-life (T_1/2z_) of approximately 2.74 ± 0.56 h, a time to peak concentration (T_max_) of about 0.79 ± 0.10 h, a peak concentration (C_max_) of 34.66 ± 5.54 ng/mL, an area under the concentration–time curve (AUC_0-t_) of 126.39 ± 12.62 ng·h/mL, and a mean residence time (MRT_0-t_) of approximately 3.73 ± 0.47 h (Qu et al., 2020). These data collectively indicate that SAC is rapidly but poorly absorbed after oral administration, with a moderate elimination rate *in vivo*. Among the three components, SAC has the lowest oral bioavailability, accompanied by insufficient renal distribution and low local exposure, which seriously restricts its application and long-term efficacy in chronic kidney disease models. Even at relatively high doses, the effective concentration reaching renal tissue is difficult to sustain, leading to weak overall intervention effects.

Currently, systematic safety assessments for SAC remain notably insufficient, lacking acute, subchronic toxicity, genotoxicity and clinical safety data. This major gap has become a key bottleneck restricting its clinical translation. Without complete safety evaluation, further development and application of SAC cannot be carried out. Therefore, future studies must involve a series of *in vitro* and *in vivo* experiments to systematically evaluate its safety profile, dose range and potential adverse effects. The pharmacokinetic profiles of SAA, SAB, and SAC indicate low oral bioavailability and rapid intestinal metabolism, including T_1/2_, T_max_, C_max_, AUC and MRT (Table 1). Therefore, it is imperative to employ emerging technologies to optimize these compounds and enhance their bioavailability. In terms of drug delivery, formulation optimization strategies have garnered significant attention to improve the bioavailability and delivery efficiency of SAA, SAB, and SAC. The development of novel delivery systems, such as nanoparticles, injectable hydrogels, and core-shell nanofibers, shows promise in enhancing their stability and bioavailability (Cheng et al., 2025; Shao et al., 2025). Kidney-targeted nanodelivery systems can specifically enrich salvianolic acids in renal tissue, prolong local action time, compensate for the defects of low bioavailability and rapid metabolism, and significantly enhance renoprotective efficacy, especially for chronic kidney injury. Additionally, structural modification of SAB is considered an effective strategy for optimizing its pharmacokinetic properties. Furthermore, to enhance the medicinal value of salvianolic acids, various extraction and purification techniques have been developed, including water extraction followed by alcohol precipitation, macroporous resin adsorption, ultrasonic-assisted extraction, high-speed countercurrent chromatography, subcritical water extraction, and supercritical CO_2_ extraction. These methods provide technical support for their large-scale production and quality control (Li et al., 2024; Qin et al., 2024; Shao et al., 2025; Wang L. et al., 2023).

**TABLE 1:** Pharmacokinetic properties of different salvianolic acids.

Drug	Animal	Route	Dose	T_1/2_ (h)	Tmax (h)	Cmax (ng/mL)	AUC_0-t_ (ng*h/mL)	MRT_0-t_ (h)	References
SAA	healthy subjects	Intravenous	10 mg/kg	1.67	—	643	148	—	Chen et al. (2022b)
SAA	healthy subjects	Intravenous	20 mg/kg	1.73	—	599	586	—	Chen et al. (2022b)
SAA	healthy subjects	Intravenous	40 mg/kg	2.2	—	1,124	1,114	—	Chen et al. (2022b)
SAA	healthy subjects	Intravenous	80 mg/kg	2.83	—	2,770	2,932	—	Chen et al. (2022b)
SAA	healthy subjects	Intravenous	120 mg/kg	1.62	—	3,925	4,101	—	Chen et al. (2022b)
SAA	healthy subjects	Intravenous	160 mg/kg	2.55	—	5,492	5,550	—	Chen et al. (2022b)
SAA	healthy subjects	Intravenous	200 mg/kg	2.26	—	9,344	9,530	—	Chen et al. (2022b)
SAA	healthy subjects	Intravenous	250 mg/kg	2.39	—	12,730	12,710	—	Chen et al. (2022b)
SAA	healthy subjects	Intravenous	300 mg/kg	2.92	—	14,310	14,660	—	Chen et al. (2022b)
SAA	Wistar rats	—	—	T_1/2z_: 3.73 ± 0.47	3.73 ± 0.47	399.25 ± 36.16	1961.37 ± 200.01	5.87 ± 0.31	Qu et al. (2020)
SAA	rats	Oral	5 mg/kg	1.96	—	31.53	105.93	—	Sun et al. (2018)
SAA	rats	Oral	10 mg/kg	1.72	—	57.39	167.18	—	Sun et al. (2018)
SAA	rats	Oral	20 mg/kg	1.79	—	111.91	317.11	—	Sun et al. (2018)
SAA	rats	Intravenous	50 μg/kg	6.16	—	578.89	204.57	​	Sun et al. (2018)
SAA	Dogs	Oral	5 mg/kg	3.25 ± 3.13	—	14.38 ± 9.12	38.77 ± 40.04	—	Sun et al. (2013)
SAA	Dogs	Oral	20 mg/kg	3.00 ± 0.79	—	38.18 ± 19.08	130.33 ± 95.53	—	Sun et al. (2013)
SAA	Dogs	Intravenous	50 μg/kg	0.05 ± 0.00	—	186.94 ± 51.45	22.17 ± 6.59	—	Sun et al. (2013)
SAA	CHF rats	Oral	772 mg/kg	17.88 ± 3.88	1.00 ± 0.00	5.20 ± 1.50	10.40 ± 1.59	—	Zhang et al. (2018a)
SAA	MCAO rats	Intravenous	2.5 mg/kg	T_1/2z_: 0.085 ± 0.059	—	29.781 ± 0.047	5.816 ± 0.129	0.118 ± 0.083	Zhao et al. (2019)
SAA	MCAO rats	Intravenous	10 mg/kg	T_1/2z_: 0.504 ± 0.013	—	132.347 ± 0.121	20.91 ± 0.048	0.199 ± 0.029	Zhao et al. (2019)
SAB	CHF rats	Oral	3,987 mg/kg	25.88 ± 1.44	1.00 ± 0.00	11.09 ± 3.17	67.54 ± 7.83	—	Zhang et al. (2018a)
SAB	MCAO rats	Intravenous	16 mg/kg	T_1/2z_: 0.03 ± 0.011	—	55.031 ± 0.151	5.783 ± 0.27	0.034 ± 0.031	Zhao et al. (2019)
SAB	Wistar rats	—	—	T_1/2z_: 3.44 ± 0.44	0.45 ± 0.13	81.68 ± 19.39	318.44 ± 39.17	4.03 ± 1.04	Qu et al. (2020)
SAB	SD rats	Intravenous	4 mg/kg	T_1/2α_: 0.139 ± 0.035 T_1/2β_: 1.346 ± 0.307	—	—	25.142 ± 6.858	1.145 ± 0.391	Hou et al. (2007)
SAB	SD rats	Intravenous	100 mg/kg	1.75 ± 0.32	—	910 ± 380	83,833 ± 9,417	—	Wu et al. (2006)
SAB	SD rats	Oral	500 mg/kg	4.13 ± 0.18	—	1.5 ± 0.5	9,700 ± 3,700	—	Wu et al. (2006)
SAB	SD rats	Intravenous	10 mg/kg	2.27 ± 1.01	—	—	11,700 ± 1,410	0.515 ± 0.225	Wu et al. (2006)
SAB	SD rats	Intravenous	50 mg/kg	1.72 ± 0.55	—	—	82,716 ± 18,133	0.53 ± 0155	Wu et al. (2006)
SAB	SD rats	Oral	50 mg/kg	2.13 ± 0.92	0.75 ± 0.77	8.93 ± 19.6	4,120 ± 8,130	—	Kim et al. (2005)
SAB	SD rats	Intravenous	100 mg/kg	T_1/2β_: 0.88 ± 0.25	—	—	22,330 ± 2,780	—	Kim et al. (2005)
SAB	SD rats	Intravenous	100 mg/kg	T_1/2β_: 0.15 ± 0.017	—	85.2 ± 12.7	34,667 ± 4,633	—	Chen et al. (2005)
SAB	rabbits	Intravenous	2.62 mg/kg	2.57 ± 1.38	—	—	2,658 ± 837	—	Ma and Wang (2007)
SAB	rabbits	Intravenous	5.25 mg/kg	3.07 ± 1.86	—	—	5,478 ± 1,670	—	Ma and Wang (2007)
SAB	rabbits	Oral	105 mg/kg	7.08 ± 2.47	2.50 ± 1.73	645 ± 450	5,135 ± 788	—	Ma and Wang (2007)
SAB	Wistar rats	Intravenous	4 mg/kg	T_1/2α_: 0.205 ± 0.036 T_1/2β_: 2.13 ± 0.078	—	—	1,463 ± 182	2.283 ± 0.062	Du et al. (2020)
SAB	Wistar rats	Intravenous	10 mg/kg	T_1/2α_: 0.378 ± 0.072 T_1/2β_: 0.27 ± 0.51	—	—	18,833 ± 5,483	2.35 ± 0.39	Du et al. (2020)
SAB	Wistar rats	Oral	10 mg/kg	—	0.333 ± 0.091	0.041 ± 0.007	21 ± 6	—	Zhang et al. (2004)
SAB	Dogs	Intravenous	3 mg/kg M	T_1/2α_: 0.37 ± 0.003 T_1/2β_: 0.72 ± 0.15	—	—	1817 ± 400	0.272 ± 0.063	Li et al. (2004)
SAB	Dogs	Intravenous	6 mg/kg M	T_1/2α_: 0.045 ± 0.008 T_1/2β_: 0.7 ± 0.12	—	—	4,133 ± 917	—	Li et al. (2004)
SAB	Dogs	Intravenous	12 mg/kg	T_1/2α_: 0.048 ± 0.005 T_1/2β_: 0.7 ± 0.17	—	—	9,700 ± 1,400	—	Li et al. (2004)
SAB	Monkeys	Intravenous	2.5 mg/kg	T_1/2_: 0.474 ± 0.110	—	28.343 ± 6.426	3.316 ± 0.871	—	Song et al. (2018)
SAB	Monkeys	Intravenous	5 mg/kg	T_1/2_: 0.540 ± 0.331	—	45.679 ± 12.301	5.754 ± 2.150	—	Song et al. (2018)
SAB	Monkeys	Intravenous	10 mg/kg	T_1/2_: 0.421 ± 0.236	—	113.293 ± 24.360	13.761 ± 2.825	—	Song et al. (2018)
SAC	Wistar rats	—	—	T_1/2z_: 2.74 ± 0.56	0.79 ± 0.10	34.66 ± 5.54	126.39 ± 12.62	3.73 ± 0.47	Qu et al. (2020)
SAC	rats	Intravenous	0.25 mg/kg	T_1/2z_: 2.988 ± 0.538	—	6,591.921 ± 1752.797	1,679.522 ± 497.306	0.578 ± 0.108	Song et al. (2016)
SAC	rats	Intravenous	0.5 mg/kg	T_1/2z_: 2.110 ± 0.878	—	14,083.521 ± 1990.136	3,089.400 ± 972.424	0.437 ± 0.109	Song et al. (2016)
SAC	rats	Intravenous	1 mg/kg	T_1/2z_: 1.887 ± 0.379	—	24,652.073 ± 4,549.767	7,512.401 ± 1,101.410	0.564 ± 0.101	Song et al. (2016)
SAC	rats	Oral	4 mg/kg	T_1/2z_: 8.169 ± 2.854	0.194 ± 0.114	25.898 ± 4.150	89.594 ± 11.606	3.257 ± 0.219	Song et al. (2016)
SAC	rats	Oral	10 mg/kg	T_1/2z_: 2.353 ± 0.359	0.458 ± 0.394	47.892 ± 7.033	212.879 ± 30.823	3.395 ± 0.183	Song et al. (2016)
SAC	rats	Oral	20 mg/kg	T_1/2z_: 1.492 ± 0.252	0.604 ± 0.329	171.483 ± 9.422	319.145 ± 41.301	3.285 ± 0.168	Song et al. (2016)

## 3 Mechanisms of SAA in different kidney diseases

### 3.1 Mechanisms of SAA in AKI

AKI is a severe clinical complication characterized by a rapid decline in renal function, leading to increased morbidity and mortality (Hu et al., 2024; Liu et al., 2020). Approximately 30%–70% of AKI patients progress to CKD or end-stage renal disease (Saranya and Viswanathan, 2023). The pathological process of AKI involves sublethal cellular injury, cell death, and inflammatory responses. Persistent inflammation following acute injury may lead to maladaptive repair, thereby promoting an unfavorable progression of AKI (Sun et al., 2019). Given the high incidence and mortality associated with AKI, there is an urgent need to develop novel drug targets and innovative therapeutic strategies. In this context, exploring the modulation of inflammation-induced injury as a potential approach to mitigate AKI holds promising prospects.

#### 3.1.1 SAA targeting peritubular capillaries (PTCs) pathway

Renal ischemia/reperfusion injury (I/R) is a primary cause of AKI, characterized by the rarefaction of peritubular capillaries (PTCs). PTCs are crucial for renal blood flow and oxygenation (Lin et al., 2025; Yang et al., 2018). This capillary rarefaction, driven by oxidative stress and excessive reactive oxygen species (ROS), leads to endothelial cell loss, inflammatory responses, cell death, and vascular damage (Xiang et al., 2019; Yang et al., 2018). In an I/R-induced AKI model, treatment with various concentrations of SAA reduced serum levels of blood urea nitrogen (BUN) and creatinine, and urinary KIM-1, alleviated renal histological injury, and preserved the integrity and density of peritubular capillary endothelial cells, thereby mitigating renal tissue hypoxia (Zhang Z. et al., 2018). Moreover, SAA inhibited platelet activation while promoting the expression of klotho protein and vascular endothelial growth factor A. Its protective mechanism is likely associated with the preservation of PTC endothelial cells, maintenance of PTCs integrity, and improvement of renal hypoxia (Zhang Z. et al., 2018). SAA also reduced renal index and pathological damage in AKI rats, lowered levels of KIM-1, NGAL, and urinary protein (UP), and serum concentrations of Scr, UREA, IL-6, IL-12, and MDA. These effects contribute to alleviating oxidative stress and protecting PTCs’ integrity (Diao et al., 2023).

#### 3.1.2 SAA targeting NF-κB/IκBα pathway

The p65 subunit of nuclear factor-κB (NF-κB) acts as a transcription factor involved in various cellular processes and plays a significant role in inflammation, immunity, cell migration, and apoptosis, all of which are associated with renal tubular cell apoptosis and the progression of AKI (Huang et al., 2025). In sepsis-associated AKI, the pathogenesis involves excessive activation of NF-κB, leading to IκBα stabilization and thereby triggering intense inflammatory responses (Cai et al., 2025). SAA has been shown to alleviate renal histopathological damage, reduce proteinuria, hypoalbuminemia, and hyperlipidemia, and mitigate oxidative stress. Additionally, SAA restores the expression of podocin, downregulates NF-κB p65 and phosphorylated IκBα (p-IκBα), and upregulates IκBα protein expression, ultimately delaying the progression of AKI (Fan et al., 2015).

#### 3.1.3 SAA targeting TLR4/MyD88 pathway

Following kidney injury and dysfunction induced by conditions such as I/R and sepsis, the levels of pro-inflammatory cytokines are significantly elevated (Hu et al., 2024). Upon stimulation by lipopolysaccharide (LPS), the membrane-embedded Toll-like receptor 4 (TLR4) is recognized and activated. The signaling of TLR4 is mediated by the adaptor protein myeloid differentiation primary response gene 88 (MyD88), which triggers a cascade of downstream inflammatory pathways, ultimately leading to the release of pro-inflammatory cytokines (Hu et al., 2024). SAA has been shown to ameliorate LPS-induced renal impairment in mice, lowering plasma creatinine and urea nitrogen levels and reducing renal tissue damage. Its mechanism involves inhibiting macrophage infiltration, decreasing the release of inflammatory factors such as TNF-α and IL-6, and downregulating the expression of COX-2 and iNOS (Zeng et al., 2020). Further studies indicate that SAA can bind to the TLR4 receptor, reduce endoplasmic reticulum stress and reactive oxygen species production, and suppress the expression of MyD88, p-PERK, CHOP, p-eIF2α, p-p38, p-ERK, and p-JNK, thereby inhibiting inflammatory responses (Zeng et al., 2020). These findings suggest that SAA may exert its anti-inflammatory effects through the TLR4/MyD88 pathway, positioning it as a potential therapeutic agent for the prevention of AKI.

#### 3.1.4 SAA targeting Akt/mTOR pathway

Studies have shown that inhibiting the protein kinase B (Akt)-mammalian target of rapamycin (mTOR) signaling pathway can alleviate inflammation, mitochondrial damage, and apoptosis induced by I/R stress, while promoting autophagy to maintain mitochondrial homeostasis, thereby protecting against AKI (Ouyang et al., 2023; Yingjie et al., 2019; Yu et al., 2023). SAA reduced plasma creatinine and urea nitrogen levels, attenuated renal damage, and decreased reactive oxygen species levels in a dose-dependent manner. This protective effect is associated with activation of the Akt/mTOR signaling pathway, as evidenced by increased protein expression of phosphorylated Akt, mTOR, and 4EBP1 (Hrbek et al., 1985). The beneficial effects could be blocked by pathway inhibitors such as LY294002 and rapamycin. Therefore, SAA may mitigate renal I/R injury through the Akt/mTOR/4EBP1 pathway, promoting the survival of renal tubular epithelial cells and representing a potential candidate for the prevention of ischemic renal injury (Hrbek et al., 1985).

### 3.2 Mechanisms of SAA in CKD

Chronic kidney disease (CKD) is a syndrome characterized by chronic renal dysfunction, involving the loss of nephrons, inflammatory responses, activation of myofibroblasts, and the deposition of extracellular matrix (ECM) (Yuan et al., 2022). CKD is a pervasive yet often silent epidemic characterized by its progressive nature. This condition is a major contributor to the development of end-stage renal disease, cardiovascular comorbidities, cachexia, and anemia. Annually, it is responsible for nearly 1.2 million deaths worldwide, imposing a significant economic burden on both affected individuals and society at large (Jankowski et al., 2021; Sridhar et al., 2025; Wang et al., 2023a).

#### 3.2.1 SAA targeting MAPK pathway

The mitogen-activated protein kinase (MAPK) signaling pathway functions as a key intracellular signal transduction system involved in the initiation, maintenance, and resolution of inflammatory responses. When resident renal cells are injured, they release pro-inflammatory cytokines and chemokines, which recruit immune cells, such as macrophages from the bone marrow. The MAPK signaling pathway serves as a primary mediator of the inflammatory interplay between these recruited immune cells and the damaged resident kidney cells (Han et al., 2025; Mohiti et al., 2025; Yuan et al., 2022). Using a 5/6 nephrectomy (Nx) rat model, SAA was shown to reduce urinary protein, serum creatinine, and other biochemical markers in a dose-dependent manner, while also alleviating renal pathological damage and fibrosis. The underlying mechanism may involve inhibition of the NF-κB and p38 MAPK signaling pathways, leading to decreased expression of inflammatory cytokines such as TNF-α, IL-1β, and MCP-1, thereby exerting a renoprotective effect (Zhang H. F. et al., 2018). Moreover, SAA reduced the phosphorylation levels of ERK 1/2, p38, JNK, and Smad 2/3, and the expression of TLR-4 and Smad 7 in CKD rats. By inhibiting the release of IL-6 and IL-1β/IL-12, decreasing MDA levels, and increasing total superoxide dismutase (T-SOD) activity, SAA demonstrated anti-inflammatory and antioxidant stress effects (Diao et al., 2023). Overall, SAA significantly ameliorates renal injury in rats, and its protective mechanism is likely associated with the modulation of the MAPKs signaling pathways.

#### 3.2.2 SAA targeting TGF-β/Smad pathway

Transforming growth factor-β1 (TGF-β1) is a key mediator in CKD, with the TGF-β/Smad pathway driving renal injury and inflammation (Chen et al., 2018). In a rat CKD model, SAA treatment reduced BUN, Scr, MDA, and kidney-to-body weight ratio, while increasing creatinine clearance rate (Ccr) and SOD activity (Zhang et al., 2019). It also lowered TGF-β1 and raised BMP-7 and Smad6 in renal tissue, suggesting that SAA protects kidneys by reducing oxidative stress and modulating TGF-β1 and BMP-7/Smad6 signaling (Zhang et al., 2019).

#### 3.2.3 SAA targeting HIF-2α/DUOX1 pathway

CKD of different etiologies presents similar histopathological patterns, including glomerulosclerosis, tubular atrophy, and interstitial fibrosis. Both macrovascular and microvascular disorders contribute to tissue ischemia, which in turn activates hypoxia-inducible factors (HIFs). HIF-2α is notably activated in CKD and is associated with depletion of nicotinamide adenine dinucleotide (NAD) in renal cells, including DUOX1 (Curran and Kopp, 2023). Exposure to sodium arsenite (NaAsO_2_) upregulates the expression of DUOX1 in renal tissues and HK-2 cells, thereby promoting ferroptosis, while knockdown of DUOX1 increases GPX4 expression and alleviates this process (Yang D. et al., 2024). Further research reveals that HIF-2α is activated following arsenic exposure and transcriptionally upregulates DUOX1 expression, thereby mediating ferroptosis. In this context, SAA has been found to effectively interfere with this pathway (Yang D. et al., 2024). This study elucidates the molecular mechanism by which NaAsO_2_ induces ferroptosis and renal injury through the HIF-2α/DUOX1 axis and suggests that SAA holds potential as a preventive or therapeutic agent.

#### 3.2.4 SAA targeting PDGF-C/PDGFR-α pathway

Platelet-derived growth factor C (PDGF-C), a newly identified member of the PDGF family, acts as a potent mitogenic factor for rat mesangial cells. It is constitutively expressed in the kidney and is specifically upregulated following significant injury to mesangial, visceral epithelial, and interstitial cells (Eitner et al., 2003; Eitner et al., 2002). This study investigated the protective effects of SAA, SAB, and their combination (SAA + SAB) against renal interstitial fibrosis through *in vivo* and *in vitro* (Yao et al., 2022). All three treatments improved renal function, reduced urinary protein and renal injury markers, upregulated the tight junction protein Par-3, and downregulated the fibrosis marker CTGF. The underlying mechanisms involved inhibition of the PDGF-C/PDGFR-α signaling pathway, as well as attenuation of human serum albumin (HSA)-induced apoptosis and endoplasmic reticulum stress in renal tubular epithelial cells (HK-2) (Yao et al., 2022). The study reveals a novel mechanism of the PDGF-C/PDGFR-α pathway in renal interstitial fibrosis and suggests that SAA, SAB, and their combination hold promise as potential therapeutic agents for the treatment of renal interstitial fibrosis (Yao et al., 2022).

### 3.3 Mechanisms of SAA in DN

#### 3.3.1 SAA targeting Nrf2 pathway

DN is characterized by renal tubular interstitial fibrosis induced by epithelial-mesenchymal transition (EMT) in renal tubular epithelial cells, and is recognized as a leading cause of end-stage renal disease. Nuclear factor erythroid 2-related factor 2 (Nrf2) serves as an intracellular defense system, counteracting oxidative stress through its antioxidant properties. Activation of Nrf2 improves the progression of glomerulonephritis by upregulating antioxidants and inhibiting ROS-mediated neutrophil extracellular trap (NET) formation (Ueda et al., 2024). SAA was found to improve vascular relaxation in diabetic mice. While SAA alone only moderately improved renal function and structure, it significantly reduced renal oxidative stress. This effect was associated with activation of the Nrf2 pathway, upregulation of antioxidant enzymes such as HO-1, NQO-1, and GPx-1, and inhibition of NF-κB p65 (Wu et al., 2016). Notably, the combination of SAA and metformin (MET) exhibited a stronger synergistic protective effect. This synergy likely stems from the combined action of MET-mediated AMPK pathway activation and SAA-induced activation of the Nrf2/antioxidant response element pathway, suggesting that the combined use of SAA and AMPK activators, such as glucose-lowering drugs, holds significant potential for preventing and treating diabetic complications (Wu et al., 2016).

#### 3.3.2 SAA targeting AGE/RAGE pathway

The formation of advanced glycation end products (AGEs) plays a central role in the pathogenesis of DN. The accumulation of AGE-mediated receptors (RAGE) triggers oxidative stress and inflammatory responses, representing the primary detrimental effects of AGEs on both the host and the intestinal microenvironment in conditions of diabetes and aging (Wu et al., 2021). RAGE activated by AGEs can induce reactive oxygen and nitrogen species through NADPH oxidase, leading to oxidative stress in the kidneys affected by DN and aging (Wu et al., 2021). SAA reduced urinary protein levels, improved creatinine clearance, and alleviated structural damage such as glomerular hypertrophy and mesangial matrix expansion in a rat model of DN. Mechanistic studies revealed that SAA inhibits actin cytoskeleton rearrangement via the AGEs-RhoA/ROCK pathway, thereby restoring glomerular endothelial permeability. Moreover, it mitigates oxidative stress and inflammatory responses through the AGE-Nox4 axis, as indicated by downregulation of ROS, NO, TNF-α, IL-6, and IL-1β, while also improving autophagy function (Hou et al., 2017). In summary, SAA protects glomerular endothelial function and reduces renal injury through multiple pathways, demonstrating a beneficial effect in early DN and highlighting its potential therapeutic value.

### 3.4 Mechanisms of SAA in NS

NS is a clinical condition characterized by massive proteinuria and reduced serum protein levels, resulting from various diseases, including minimal change nephrotic syndrome, focal segmental glomerulosclerosis, and membranous glomerulonephritis (Tsuji et al., 2020; Zabala Ramirez et al., 2023). This study investigated the role of soluble urokinase-type plasminogen activator receptor (suPAR) and its membrane-bound form (uPAR) in patients with steroid-resistant nephrotic syndrome (SRNS), and evaluated the potential of SAA to ameliorate podocyte steroid resistance (Li et al., 2021). The results demonstrated that serum and urinary suPAR levels were significantly higher in SRNS patients compared to healthy individuals and steroid-sensitive patients. In renal tissues, uPAR expression was negatively correlated with glucocorticoid receptor α (GRα) and positively correlated with GRβ. Combined induction with TNF-α and IFN-γ upregulated GRβ expression in podocytes, reduced the GRα/GRβ ratio, and induced a steroid-resistant state. Treatment with SAA significantly decreased podocyte apoptosis, downregulated suPAR/uPAR expression, and increased the expression of the podocyte marker protein Nephrin (Li et al., 2021). In conclusion, the expression levels of suPAR/uPAR show potential value in predicting steroid resistance in patients with primary nephrotic syndrome. Moreover, SAA may improve podocyte sensitivity to glucocorticoids by modulating the suPAR/uPAR-αvβ3 signaling pathway, providing new experimental evidence for the treatment of NS (Li et al., 2021).

### 3.5 Mechanisms of SAA in SLE

SLE is a chronic inflammatory disease, with approximately 50% of patients experiencing renal involvement. Lupus nephritis (LN) represents the most common and severe organ manifestation of SLE. A majority of patients eventually progress to chronic kidney disease or end-stage renal disease, significantly increasing morbidity and mortality (Almaani et al., 2017; Rovin, 2022). After 5 months of intervention with SAA, the levels of anti-Sm autoantibodies were significantly reduced in SLE mice, and renal pathological damage was alleviated. Mechanistic studies indicated that SAA inhibited the phosphorylation of IKK, IκB, and NF-κB in the renal tissues of lupus mice (Lin et al., 2017). These findings suggest that SAA may exert therapeutic effects on SLE by suppressing the activation of the NF-κB signaling pathway, thereby mitigating autoimmune responses and renal injury.

Compared with SAB and SAC, SAA has unique advantages in protecting peritubular capillaries and improving renal hypoxia, and shows specific efficacy in steroid-resistant nephrotic syndrome, which is a distinctive and irreplaceable feature of its renoprotective mechanism. These characteristics make SAA more suitable for renal injury accompanied by microvascular damage and steroid resistance.

**FIGURE 1:**
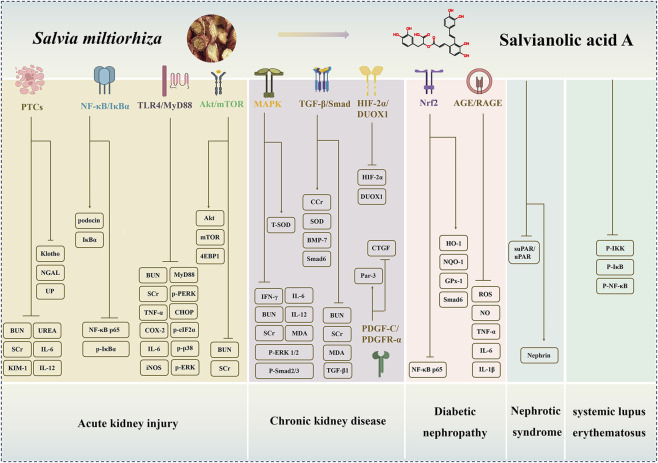
The regulatory mechanisms and renoprotective effects of Salvianolic acid A (SAA) in multiple kidney diseases. SAA exerts anti-AKI effects by mediating PTCs, NF-κB/IκBα, TLR4/MyD88, Akt/mTOR pathways; exerts anti-CKD effects by regulating MAPK, TGF-β/Smad, HIF-2α/DUOX1, PDGF-C/PDGFR-α pathways; exerts anti-DN effects via Nrf2, AGE/RAGE pathways; improves NS and SLE by targeting suPAR/uPAR-αvβ3, NF-κB pathways. (AKI: acute kidney injury; CKD: chronic kidney disease; DN: diabetic nephropathy; NS: nephrotic syndrome; SLE: systemic lupus erythematosus; PTCs: peritubular capillaries).

Preclinical studies demonstrate that SAA alleviates kidney injury in models of acute, chronic, and DN through multifaceted actions. These include attenuating inflammation and oxidative stress through pathways such as NF-κB and Nrf2, promoting cell survival by modulating Akt/mTOR and TGF-β/Smad signaling, preserving renal microcirculation, and intervening in disease-specific processes, including ferroptosis and steroid resistance (Figure 1). However, significant translational challenges remain, including the precise molecular targets of SAA are not fully defined. Furthermore, key pharmacological properties, including its bioavailability, tissue distribution, and long-term safety profile, must be thoroughly characterized to assess its therapeutic potential in humans. While SAA represents a promising multi-target agent, bridging the gap from preclinical promise to clinical application demands more rigorous and translational research.

## 4 Mechanisms of SAB in different kidney diseases

### 4.1 Mechanisms of SAB in AKI

#### 4.1.1 SAB targeting Nrf2/NLRP3 pathway

SAB has been shown to improve renal function and reduce levels of oxidative stress and inflammatory factors. Studies have found that renal I/R injury upregulates the expression of NLRP3, caspase-1, GSDMD, and IL-1β in renal tissues, while pretreatment with SAB promotes nuclear accumulation of Nrf2 and suppresses oxidative stress and inflammatory responses (Pang et al., 2020). Further mechanistic investigations indicate that SalB alleviates renal I/R injury in mice by activating the Nrf2/NLRP3 signaling pathway and inhibiting caspase-1/GSDMD-mediated pyroptosis (Pang et al., 2020).

#### 4.1.2 SAB targeting PRDX5 pathway

The expression of immediate early response gene 3 (IER3) is significantly upregulated in senescent renal tubular epithelial cells. Upon IER3 deletion, peroxiredoxin 5 (PRDX5) expression is markedly increased in AKI patients (Dou et al., 2025). Reduced PRDX5 levels have been shown to accelerate renal injury (Choi et al., 2025). In this study, using cisplatin- and folic acid-induced acute kidney injury models, SAB was found to effectively ameliorate renal dysfunction *in vivo* and improve cisplatin-induced injury in renal tubular epithelial cells. Through target prediction and validation, SAB was shown to directly bind to the PRDX5 protein and enhance its redox activity. This subsequently upregulated the expression of SLC7A11 and GPX4, thereby inhibiting cisplatin-induced ferroptosis (Tao et al., 2025). Knockdown of PRDX5 partially reversed the protective effect of SAB.

#### 4.1.3 SAB targeting PI3K/Akt pathway

Pretreatment with SAB for 7 days before I/R induction significantly reduced SCr and BUN levels, thereby improving renal function (Ma et al., 2017). SAB effectively alleviated renal oxidative stress by inhibiting the production of the lipid peroxidation product MDA and enhancing antioxidant activity. Additionally, SAB reduced the levels of inflammatory markers and neutrophil infiltration. Further mechanistic studies revealed that SAB upregulated PI3K protein expression and increased the p-Akt/Akt ratio, indicating activation of the PI3K/Akt signaling pathway (Ma et al., 2017). These findings suggest that SAB exerts renoprotective effects by modulating the PI3K/Akt pathway, thereby therapeutically mitigating oxidative stress and inflammation.

### 4.2 Mechanisms of SAB in CKD

#### 4.2.1 SAB targeting BMP2/Smads pathway

Bone morphogenetic protein 2 (BMP2) serves as a fundamental component of the intrinsic regenerative capacity of bone tissue, promoting cell migration and invasiveness *in vitro* through the activation of the Smad1/5/8 signaling pathway (Rosen, 2009; Wu et al., 2022). In a study using a rat model of chronic kidney disease with arteriovenous fistula vascular calcification (CKD-AVF), treatment with different doses of SAB reduced SCr, BUN, and alkaline phosphatase (ALP) levels. Histopathological analysis demonstrated that SAB effectively alleviated tissue damage in both the kidney and the venous segment of the arteriovenous fistula, improved renal fibrosis, and reduced calcium deposition in the venous segment. Further protein expression analysis revealed that SAB downregulated the expression of osteogenesis-related proteins in vascular tissues, including BMP-2, p-Smad1/5, Osterix, and Runx2 (Ye et al., 2025). These results indicate that SAB improves renal function and inhibits ectopic calcification, with its mechanism likely associated with suppression of the BMP-2/Smads signaling pathway.

#### 4.2.2 SAB targeting NEU1 pathway

Mutations in the Neuraminidase 1 (NEU1) gene can lead to the sudden onset of fulminant glomerulonephropathy. NEU1-deficient mouse models develop end-stage urinary retention and severe renal injury, characterized by elevated urinary protein levels, loss of nephrons, renal fibrosis, formation of storage vacuoles, and abnormal mitochondrial morphology in both glomerular and tubular cells (Kho et al., 2023). This study investigated the protective effect and mechanism of SAB in mouse models of renal injury induced by unilateral ureteral obstruction (UUO) and I/R. Treatment with SAB (40 mg/kg) alleviated renal tissue damage and fibrosis in the UUO model, and inhibited the expression of renal injury markers such as KIM-1, epithelial–mesenchymal transition-related proteins Snail-1/2, and pro-inflammatory factors (TNF-α, IL-6, and IL-1β). Additionally, SAB reduced collagen deposition and suppressed phosphorylation of ALK5 and downstream Smad2/3 in the TGF-β pathway. In the I/R model, SAB similarly demonstrated clear renoprotective effects (Chen et al., 2023). Notably, the study compared SAB with its structural analog SAA. While SAA affected some indicators (such as KIM-1 and Col1a1) at the same concentration, its effects were significantly weaker than those of SAB in inhibiting collagen deposition, Snail-2, TNF-α, and the activation of key signaling pathways (ALK5/Smad2/3). This difference in protective efficacy aligns with previous findings that SAB has a stronger binding affinity for the target NEU1 compared to SAA (Chen et al., 2023). To validate the key mediating role of NEU1, the study used NEU1 conditional knockout (CKO) mice. In these mice, all protective effects of SAB-including alleviation of pathological injury, inhibition of fibrosis and inflammation, and suppression of pathway activation-were completely abolished. This indicates that NEU1 is essential for SAB to exert its comprehensive renoprotective actions (Chen et al., 2023). In summary, this study confirms that SAB targets and binds to NEU1, effectively inhibiting the TGF-β/Smad signaling pathway and inflammatory responses, thereby exerting potent anti-injury and anti-fibrotic effects that surpass those of its structural analog SAA. NEU1 is identified as the key molecular target mediating the renal protective function of SAB.

#### 4.2.3 SAB targeting TGF-β pathway

TGF-β1 treatment for 72 h induced HK-2 cell transformation into myofibroblasts, but co-treatment with SAB reversed this morphology and restored epithelial features. This reversal was dose-dependent, with decreased α-SMA (mesenchymal marker) and increased E-cadherin (epithelial marker) (Pan et al., 2011). Similar findings were reported by Yao et al. (2009). Collectively, these results indicate that SAB inhibits TGF-β1 signaling and blocks epithelial-mesenchymal transition (EMT), highlighting its therapeutic potential for renal tubulointerstitial fibrosis by promoting repair and reversing fibrosis (Pan et al., 2011).

This study developed a kidney-targeted nanocomplex, HChi-Ca-SAB, for delivering SAB to treat renal fibrosis. The complex was constructed through the coordination assembly of catechol-modified chitosan (Li et al., 2017). The nanocomplex effectively reversed TGF-β1-induced epithelial-mesenchymal transition in human renal tubular epithelial cells. *In vivo* imaging demonstrated its favorable renal-specific distribution. In a mouse model of unilateral ureteral obstruction, the HChi-Ca-SAB nanocomplex significantly alleviated renal morphological damage and reduced extracellular matrix protein deposition compared with free SAB, showing stronger anti-fibrotic efficacy (Li et al., 2017). This result directly confirms that optimizing pharmacokinetic properties and improving renal targeting through nanodelivery systems can significantly enhance the anti-fibrotic effect of SAB, which provides a practical solution to the bottleneck of short half-life and insufficient renal exposure.

#### 4.2.4 SAB targeting Sirt1/autophagy pathway

Sirt1 is a highly conserved NAD^+^-dependent histone deacetylase involved in regulating various pathophysiological processes, including cell proliferation, survival, differentiation, autophagy, and oxidative stress (Ding et al., 2024). Enhancement of the Sirt1/autophagy axis can inhibit NLRP3 inflammasome activation and improve renal function, proteinuria, and renal pathology, demonstrating significant protective effects, especially against myocardial I/R injury (Yang et al., 2020). SAB ameliorated renal dysfunction and significantly reduced the expression of fibrosis-related proteins, such as fibronectin, α-SMA, and TGF-β *in vivo* (He et al., 2020). Furthermore, SAB upregulated the expression of Sirt1 and activated the autophagy process, as indicated by increased levels of Beclin1 and the LC3II/I ratio, alongside decreased p62 expression. *In vitro* studies further confirmed that SAB could reverse TGF-β1-induced EMT in human renal tubular epithelial cells. Mechanistic investigations revealed that inhibiting either Sirt1 or autophagy abolished the protective effect of SAB against EMT (He et al., 2020). In summary, this study demonstrates that SAB alleviates renal fibrosis by activating the Sirt1-mediated autophagy pathway, thereby inhibiting EMT. These findings highlight Sirt1 as a key therapeutic target in this protective mechanism.

#### 4.2.5 SAB targeting PTEN pathway

Phosphatase and tensin homolog (PTEN), also known as phosphatase and tensin-related phosphatase, plays a key role in regulating the physiological behavior of renal tubular epithelial cells, glomerular cells, and interstitial cells, thereby maintaining the structural and functional homeostasis of the kidney. In the transition from AKI to CKD, PTEN significantly influences cellular proliferation, apoptosis, fibrosis, and mitochondrial energy metabolism (Cao et al., 2024). SAB treatment reduced SCr and BUN levels, and interstitial collagen deposition, in mouse models of UUO and aristolochic acid nephropathy (AAN). Both *in vivo* and *in vitro* experiments demonstrated that SAB inhibited the expression of fibrosis markers FN and α-SMA (Lin et al., 2023). Mechanistic studies revealed that SAB exerts its antifibrotic effects by downregulating the expression of EZH2 and the histone modification marker H3K27me3, which in turn activates PTEN and suppresses Akt phosphorylation. The protective effect of SAB on renal fibroblasts was attenuated when EZH2 inhibitors were used (Lin et al., 2023). These findings suggest that SAB may counteract renal fibrosis in chronic kidney disease by inhibiting EZH2 expression, highlighting its potential for clinical translation.

#### 4.2.6 SAB targeting miR-106b-25 pathway

The miR-106b-25 cluster, comprising miR-25-3p, miR-93-5p, and miR-106b-5p, is located within the MCM7 gene. Studies have shown that this cluster is upregulated in renal cell carcinoma (RCC) (Gluchowska and Hofman, 2025). miRNA microarray screening revealed that the miR-106b-25 cluster is significantly downregulated during TGF-β1-induced EMT in renal tubular epithelial cells. Overexpression of cluster members, particularly miR-106b, effectively maintains epithelial morphology and reduces the mesenchymal marker α-SMA. Bioinformatics analysis and dual-luciferase reporter assays confirmed that these miRNAs directly target and inhibit the expression of TGF-β receptor type II (Tang et al., 2014). Further research demonstrated that SAB upregulates the expression of the miR-106b-25 cluster, thereby suppressing TGF-β signaling and the EMT process. Thus, the miR-106b-25 cluster not only participates in renal EMT but also mediates the anti-fibrotic protective effects of SAB, suggesting that targeting specific miRNAs represents a potential novel strategy for the treatment of renal fibrosis (Tang et al., 2014).

#### 4.2.7 SAB targeting HPSE/SDC1 pathway

Heparanase-1 (HPSE), an endoglycosidase, participates in the complex biological regulatory network underlying various proteinuric renal diseases. Its leading role in the renal fibrosis signaling pathway may significantly influence the progression of CKD (Masola et al., 2015). Syndecan-1 (SDC1), a transmembrane chondroitin sulfate proteoglycan, functions as a receptor in the ECM and is involved in intercellular communication, cell proliferation, angiogenesis, and metastasis (Song et al., 2025). SAB treatment reduced sSCr, BUN, and levels of TGF-β1 and FGF-2 in a mouse model of unilateral ureteral obstruction, while also improving renal pathological damage. SAB dose-dependently suppressed angiotensin II-induced expression of heparanase, FGF-2, TGF-β1, and α-SMA in renal tubular epithelial cells, while upregulating syndecan-1 and E-cadherin (Hu et al., 2020). These findings indicate that SAB exerts renoprotective effects by inhibiting the HPSE/SDC1 signaling axis, offering a novel potential strategy for the treatment of renal interstitial fibrosis.

### 4.3 Mechanisms of SAB in DN

#### 4.3.1 SAB targeting PI3K/Akt/NF-κB pathway

In a rat model of early-stage DN induced by streptozotocin (STZ) combined with a high-glucose and high-fat diet, the combination of SAB and tanshinone IIA synergistically reduced 24 h urinary total protein, BUN, and SCr levels, ameliorated renal pathological injury, and corrected metabolic disturbances by modulating pathways related to saturated fatty acids, glycerophospholipids, and steroids. Moreover, the combined treatment significantly attenuated renal inflammation, upregulated the p-PI3K/PI3K and p-Akt/Akt ratios, and suppressed p-NF-κB/NF-κB activation, with effects superior to monotherapy. These benefits were reversed by the PI3K inhibitor LY294002 (Xu et al., 2024). The combination of SAB and tanshinone IIA synergistically improves glucose-lipid metabolic disorders, hepatic-renal injury, and renal inflammation in early-stage DN rats, likely through the regulation of the PI3K/Akt/NF-κB signaling pathway. Furthermore, a certain dose of SAB can inhibit high-glucose-induced mesangial cell proliferation and ECM production. SAB may regulate cell-cycle progression and the activity of MMP-2 and MMP-9 by suppressing NF-κB activation, suggesting that SAB could be an effective agent for the treatment of DN (Luo et al., 2008).

#### 4.3.2 SAB targeting endoplasmic reticulum stress pathway

Metabolic stress adaptation failure disrupts endoplasmic reticulum (ER) homeostasis, thereby triggering ER stress-induced cell death in diabetic nephropathy. Clinically, markers of ER stress are associated with proteinuria and tubular injury in progressive DN (Yang et al., 2025). In mice fed a high-fat diet (HFD), renal cell apoptosis (increased Bax and cleaved caspase-3) and ER stress (upregulated markers such as BIP, p-eIF2α, ATF4, and CHOP) were induced. Palmitic acid similarly promoted apoptosis and ER stress in HK-2 cells (Mai et al., 2020). Notably, SAB was able to inhibit the apoptosis and ER stress induced by palmitic acid, but it did not suppress ER stress triggered by tunicamycin or thapsigargin (Mai et al., 2020).

#### 4.3.3 SAB targeting oxidative stress pathway

SAB alleviates renal pathological injury, improves renal function, and inhibits apoptosis in rat models of diabetic nephropathy. Its mechanisms of action include enhancing the activities of GSH and SOD and reducing the levels of ROS and MDA *in vivo* and *in vitro*. Moreover, SAB suppresses the expression of inflammatory cytokines (such as IL-1β and IL-6) and downregulates type IV collagen and fibronectin in high-glucose-stimulated glomerular mesangial cells (Wen et al., 2023). The renoprotective effects of SAB are associated with its ability to attenuate oxidative stress and activate the SIRT3/FOXO1 signaling pathway, suggesting its potential as a therapeutic agent for DN.

#### 4.3.4 SAB targeting ADORA2B pathway

Extracellular adenosine signals through ADORA2B, primarily generated via the nucleotide phosphohydrolase activity of CD73, and plays a functional role in diabetic nephropathy, during which ADORA2B is selectively induced and upregulated (Tak et al., 2014). In the db/db mouse, SAB intervention for 6 weeks improved hyperglycemia, hyperlipidemia, and renal function, while alleviating renal pathological injury. The underlying mechanism involved inhibition of adenosine A2B receptor (ADORA2B) expression, leading to the downregulation of downstream TLR4/NF-κB signaling and NALP3 activation, ultimately reducing the release of inflammatory cytokines such as interleukin-1β (Wang et al., 2024). In high-glucose-treated HK-2 cells, SAB was also shown to reduce the expression of ADORA2B, NALP3, and NF-κB, along with decreasing ROS production (Wang et al., 2024). Therefore, SAB may mitigate renal tubular injury in DN by suppressing the ADORA2B-mediated inflammatory pathway.

### 4.4 Mechanisms of SAB in membranous nephropathy (MN)

The histomorphological hallmark of membranous nephropathy (MN) is the presence of immune deposits in the subepithelial space of the glomerular filtration barrier. Clinically, it is characterized by nephrotic-range proteinuria with edema. Despite the efficacy of B-cell depletion therapy, many MN patients only achieve partial remission of proteinuria (Hoxha et al., 2022; Kistler and Salant, 2024). In a rat model of MN induced by cationic bovine serum albumin, SAB treatment reduced 24 h urinary protein, SCr, and BUN levels, ameliorated renal pathological injury, decreased glomerular deposition of CD68 and IgG, and promoted autophagosome formation in the mesangial area (Chen et al., 2022a). Additionally, SAB alleviated lipopolysaccharide-induced proliferation of human mesangial cells (HMCs), lowered the expression of inflammatory cytokines (IL-1β, IL-6, and TNF-α), and activated the autophagy-related protein beclin 1. Mechanistically, SAB upregulated the expression of microRNA-145-5p, which in turn suppressed the activation of the PI3K/Akt signaling pathway. The protective effects of SAB were reversed by either a microRNA-145-5p inhibitor or the PI3K inhibitor LY294002 (Chen et al., 2022a). SAB likely protects against membranous nephropathy by upregulating microRNA-145-5p, inhibiting the PI3K/Akt pathway, and subsequently activating renal autophagy, reducing mesangial cell proliferation and inflammatory responses.

### 4.5 Mechanisms of SAB in contrast-induced nephropathy (CIN)

With the widespread use of contrast agents in medical imaging, contrast-induced nephropathy (CIN) has become the third leading cause of iatrogenic renal dysfunction. This study investigated the protective effect and mechanism of SAB against iopromide-induced injury in human renal tubular epithelial cells. The results showed that SAB, at various concentrations, counteracted iopromide-induced apoptosis, reduced intracellular ROS levels, and downregulated the expression of ER stress-related proteins and apoptosis-associated proteins, including p-IRE-1α, p-eIF-2α/eIF-2α, p-JNK, CHOP, and Bax/Bcl-2 (Dong et al., 2021). At a concentration of 100 μmol/L, SAB inhibited ER stress and alleviated cellular injury to an extent similar to that of the ER stress inhibitor 4-PBA. Moreover, SAB was able to reverse the damage induced by the ER stress agonist tunicamycin. SAB exerted its protective effects by increasing cell viability and mitochondrial membrane potential, lowering ROS levels, and downregulating the expression of pro-apoptotic and key ER stress-related proteins (Dong et al., 2021). These findings suggest that the protective effect of SAB against CIN injury is closely associated with the inhibition of the ER stress pathway.

### 4.6 Mechanisms of SAB in RCC

Globally, RCC ranks as the sixth most common cancer in men and the tenth in women, accounting for approximately 5% and 3% of all tumor diagnoses, respectively, with its incidence rising annually (Capitanio et al., 2019). Low shear stress-a type of fluid mechanical stimulus-can induce the activation and nuclear accumulation of Yes-associated protein 1 (YAP1) in RCC cells, promoting EMT and enhancing resistance to anoikis, which may contribute to tumor metastasis (Chen X. et al., 2022). Inhibition of ROCK or RhoA signaling partially blocks YAP1 nuclear accumulation. Further research demonstrated that SAB effectively reverses low shear stress-induced EMT by suppressing YAP1 activation and its downstream Hippo signaling pathway. These findings suggest that YAP1 acts as a mechanical sensor that converts low shear stress into pro-tumorigenic signals, while SAB exhibits potential to counteract mechanically driven tumor progression by targeting the YAP1/Hippo pathway (Chen X. et al., 2022).

SAB has stronger target binding ability (such as NEU1) and anti-fibrotic efficacy than SAA and SAC, and can regulate multiple pathways at the epigenetic and miRNA levels, showing comprehensive advantages in renal fibrosis and chronic kidney injury. This is closely related to its high target affinity and multi-level regulatory mechanism.

**FIGURE 2:**
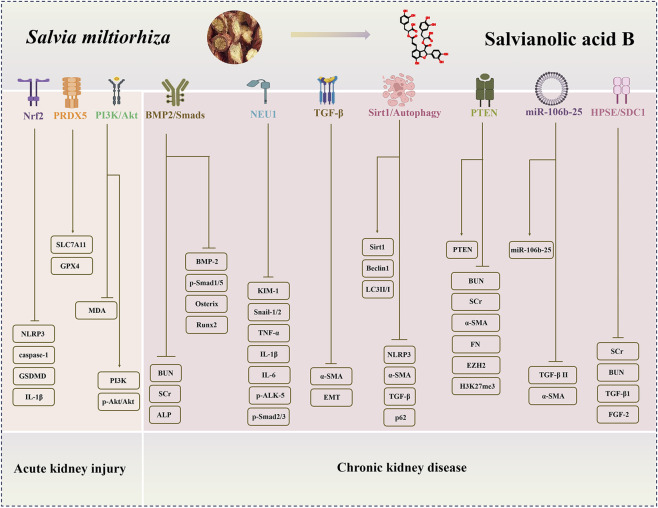
The regulatory mechanisms and renoprotective effects of Salvianolic acid B (SAB) in AKI and CKD. SAB alleviates AKI through Nrf2/NLRP3, PRDX5, PI3K/Akt pathways; ameliorates CKD by regulating BMP2/Smads, NEU1, TGF-β, Sirt1/autophagy, PTEN, miR-106b-25, HPSE/SDC1 pathways. (AKI: acute kidney injury; CKD: chronic kidney disease).

**FIGURE 3:**
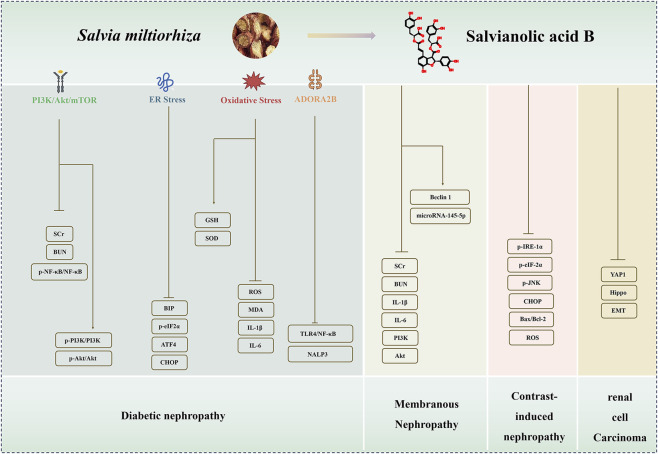
The regulatory mechanisms and renoprotective effects of Salvianolic acid B (SAB) in DN, MN, CIN and RCC. SAB exerts anti-DN effects via PI3K/Akt/NF-κB, endoplasmic reticulum stress, SIRT3/FOXO1, ADORA2B pathways; protects against MN, CIN and RCC by targeting PI3K/Akt, endoplasmic reticulum stress, YAP1/Hippo pathways. (DN: diabetic nephropathy; MN: membranous nephropathy; CIN: contrast-induced nephropathy; RCC: renal cell carcinoma).

Research indicates that SAB exerts multiple protective effects, including anti-inflammatory, antioxidant, and antifibrotic activities, as well as inhibition of pyroptosis and ferroptosis, by modulating key targets and pathways such as Nrf2/NLRP3, PI3K/Akt, TGF-β/Smad, Sirt1/autophagy, and NEU1 (Figures 2, 3). It also demonstrates potential in reversing epithelial–mesenchymal transition and improving renal microcirculation. Notably, pharmacokinetic studies in chronic kidney disease models reveal increased renal exposure of its active metabolite, Danshensu, providing a pharmacokinetic rationale for its mechanism of action. However, current evidence is largely derived from preclinical studies, and its efficacy in humans, optimal dosing regimens, and long-term safety remain to be validated in clinical trials. Reported mechanisms vary across different models, and their precise primary molecular target(s) require further clarification. Additionally, SAB exhibits low bioavailability. Although renal-targeted delivery systems have been explored, their clinical translation still faces challenges. In summary, SAB is a multi-target candidate drug with promising therapeutic potential, but further translational research is essential to advance its clinical application.

## 5 Mechanisms of SAC in different kidney diseases

### 5.1 Mechanisms of SAC in AKI

Pretreatment with SAC ameliorated cisplatin-induced renal pathological damage, reduced Scr and BUN levels, and alleviated oxidative stress and inflammation. Specifically, it lowered renal levels of MDA and inflammatory mediators such as iNOS, COX-2, TNF-α, IL-6, and IL-1β, while elevating glutathione (GSH) and the activities of multiple antioxidant enzymes including superoxide dismutase 1 and glutathione peroxidase 3 (Chien et al., 2021). Mechanistic studies revealed that SAC acts by modulating several signaling pathways: it inhibited the activation of NF-κB and the phosphorylation of mitogen-activated protein kinases, while downregulating TLR4 expression. Furthermore, SAC enhanced the antioxidant defense function of Nrf2 and its downstream target heme oxygenase-1, and upregulated the expression of the deacetylase Sirtuin 1. Notably, the protective effects of SAC were reversed by the Sirtuin 1-specific inhibitor EX-527 (Chien et al., 2021). Thus, SAC likely protects against cisplatin-induced nephrotoxicity by activating Sirtuin 1 and coordinately regulating the Nrf2 antioxidant pathway and the NF-κB inflammatory pathway, thereby mitigating oxidative stress and inflammation. Compared with SAA and SAB, SAC’s anti-inflammatory and antioxidant effects are more dependent on Sirtuin1 pathway activation, showing obvious pathway specificity. This is a distinctive feature of SAC, but also leads to its relatively single regulatory mode and weak overall efficacy. These findings offer a potential therapeutic target for the clinical prevention and treatment of cisplatin-related renal injury.

### 5.2 Mechanisms of SAC in CKD

SAC alleviates the extent of renal interstitial fibrosis and inhibits the accumulation of extracellular matrix proteins. Mechanistic studies have shown that SAC downregulates the expression of EMT-related factors and its key transcription factor, Snail. Further experiments confirmed that SAC blocks the EMT process by suppressing the activation of the TGF-β/Smad signaling pathway (Wu et al., 2023). However, SAC’s anti-fibrotic efficacy is weaker than that of SAB, and its regulation intensity of TGF-β/Smad pathway is lower than SAA and SAB. SAC mitigate renal tubulointerstitial fibrosis primarily by inhibiting EMT through the modulation of the TGF-β/Smad pathway, without involving multi-level regulation such as epigenetics and miRNAs.

At present, there is a lack of direct research on SAC in DN models. Based on its antioxidant and anti-inflammatory properties, SAC may exert a renoprotective effect by regulating oxidative stress and inflammatory response in DN, but its specific targets and pathways need to be further verified. Similarly, research on SAC in NS, LN and other kidney diseases is completely blank, which is an important direction for future research. Research on SAC in kidney disease is relatively focused, with weak evidence in pharmacokinetics, safety and multi-disease models. Its protective effect is concentrated on anti-inflammatory, antioxidant and anti-fibrotic aspects, with pathway specificity but low efficacy intensity, and its translational potential needs more basic research support.

## 6 Clinical translation challenges and optimization strategies

Although salvianolic acids show significant renoprotective effects in preclinical studies, their clinical translation faces multiple key bottlenecks, which are closely related to their inherent physicochemical and pharmacokinetic properties, as well as insufficient research evidence. SAA and SAB have completed preliminary animal and human safety evaluations, but still lack long-term, large-sample, high-quality clinical safety data, especially in patients with kidney disease. For SAC, systematic safety evaluation is almost blank, including acute toxicity, subchronic toxicity, genotoxicity, reproductive toxicity and clinical safety, which has become a core obstacle to translation. All three components have common pharmacokinetic problems: low oral bioavailability, rapid *in vivo* metabolism, short half-life, and insufficient renal tissue distribution. These characteristics lead to low effective concentration in renal tissue, difficulty in sustaining long-term efficacy, especially for chronic progressive kidney diseases such as CKD and renal fibrosis, which seriously affect the therapeutic effect.

The direct molecular targets of SAA and SAC have not been fully clarified; most mechanisms are still at the pathway association level. The research evidence of SAC is extremely insufficient, limited to AKI and CKD models, and lacks verification in DN, NS, LN and other diseases. The overall evidence chain is incomplete, which affects the accuracy and predictability of clinical application. Although kidney-targeted nanodelivery systems have shown good effects in preclinical studies, they still face problems such as a complex preparation process, high cost, difficult scale-up production, and unclear long-term biosafety, which limit clinical transformation and application. At present, the research on salvianolic acids in kidney diseases is still in the preclinical stage, and there is a lack of large-scale, randomized, double-blind, placebo-controlled clinical trials to confirm their efficacy and safety in patients, resulting in unclear human dosage regimens and administration strategies. To overcome the above bottlenecks, future research should focus on the following strategies: develop kidney-targeted nanodelivery systems to improve bioavailability, renal specific distribution and local retention time, thereby enhancing therapeutic efficacy; carry out structural modification of salvianolic acids to improve stability, target affinity and pharmacokinetic properties; systematically evaluate the safety of SAC, and carry out long-term clinical safety studies of SAA and SAB; clarify the direct molecular targets of the three components, and fill the research gaps of SAC in DN, NS and other diseases; explore the synergistic effect of salvianolic acids combined with Traditional Chinese medicine or western medicine to improve therapeutic efficacy; carry out well-designed clinical trials to confirm the efficacy and safety in kidney disease patients.

## 7 Discussion

This review summarizes the pharmacological effects and mechanistic advances of SAA, SAB, and SAC in various kidney diseases, revealing the significant potential of these natural products to provide renal protection through multi-target and multi-pathway synergistic actions. Overall, although SAA, SAB, and SAC share structural similarities, they exhibit certain differences in mechanisms of action, target affinity, and pharmacological potency, which offer possibilities for precise intervention in kidney diseases with distinct pathological features.

All three salvianolic acids act on core pathological processes in renal diseases. In terms of antioxidant effects, each activates the Nrf2/ARE pathway, upregulates antioxidant enzymes such as HO-1 and SOD, and reduces oxidative damage markers like MDA. Regarding anti-inflammatory actions, they inhibit the activation of key inflammatory pathways, including NF-κB and MAPK, and decrease the release of pro-inflammatory cytokines such as TNF-α and IL-6. Particularly in anti-fibrotic effects, SAA, SAB, and SAC each interfere with the TGF-β/Smad signaling axis, inhibit the EMT process, and reduce extracellular matrix deposition. Among them, SAB has the strongest regulatory effect on TGF-β/Smad pathway and EMT reversal, followed by SAA, and SAC is the weakest; SAA is unique in protecting peritubular capillaries and improving renal hypoxia; SAC relies on Sirtuin1 to coordinate antioxidant and anti-inflammatory responses, showing obvious pathway dependence. However, further comparative analysis reveals that SAB demonstrates stronger efficacy in suppressing TGF-β signaling and reversing EMT, which may be related to its higher affinity for specific targets such as neuraminidase 1 (NEU1). In contrast, Salvianolic Acid A shows unique advantages in protecting the integrity of peritubular capillaries and improving tissue hypoxia. SAC functions by activating Sirtuin1 and coordinately modulating the Nrf2 and NF-κB pathways, thereby playing a role in balancing antioxidant and anti-inflammatory responses (Table 2).

**TABLE 2:** Mechanisms of different salvianolic acids in different kidney disease.

Drug	Disease	*In vivo*/*vitro*	Animal/cell model	Dosage	Duration	Mode of administration	Described effects	Pathways	References
SAA	AKI	*In vivo*	Renal ischaemia-reperfusion (I/R) injury-induced male SD rats	2.5, 5, 10 mg/kg	24 h	Gavage	↑: VEGFA ↓: BUN, SCr, KAM-1, ATN, HIF-1α, vWFL, CD61/CD62P, Klotho	PTC integrity	Zhang et al. (2018c)
	​	*In vivo*	Gentamicin-induced SD rats	10, 20, 40 mg/kg	7 days	Gavage	↓: KAM-1, NGAL, UP, MDA	Anti-oxidative stress	Diao et al. (2023)
	​	*In vivo*	DOX-induced male SD rats	2.5, 5, 10 mg/kg	4 weeks 2 h	Intravenously	↑: ALB, SOD, podocin, IκBα ↓: TG, TC, BUN, SCr, MDA, p-IκBα, p-NF-κB	NF-κB, IκBα	Fan et al. (2015)
	​	*In vivo and* *In vitro*	LPS-induced male BALB/C mice Raw264.7 cell	60 mg/kg 0.1, 1, 10, 100 μM	12 h 24 h	Gavage —	↓: BUN, SCr, IL-6, TNF-α, iNOS, COX-2, TLR4, MyD88, p-PERK, CHOP, p-eIF2α, p-p38, p-ERK, p-JNK	TLR4/MyD88	Zeng et al. (2020)
	​	*In vivo and* *In vitro*	Renal ischaemia-reperfusion (I/R) injury-induced male SD rats HK cell	40 mg/kg 10, 20, 40 μM	24 h 2.5 h	Intravenously —	↑: SOD, p-Akt, p-mTOR, p-4EBP1 ↓: BUN, SCr, Bcl-2, Cleaved caspase 3, MDA, ROS	Akt/mTOR/4 EBP 1	Hrbek et al. (1985)
	CKD	*In vivo and* *In vitro*	5/6 nephrectomized (5/6Nx) rats HK cell	2.5, 5, 10 mg/kg 3, 10, 30 μM	4 weeks 2 h	intraperitoneally	↓: BUN, SCr, TC, TG, TGF-β, α-SMA, TNF-α, IL-1β, MCP-1, KAM-1, p-IKKα/β, p-NF-κB, p-p38 MAPK, ICAM-1, VCAM-1	NF-κB, p38 MAPK	Zhang et al. (2018b)
	​	*In vivo*	5/6 nephrectomy (5/6Nx) -induced SD rats	10, 20, 40 mg/kg	4 weeks 2 h	Gavage	↓: UREA, SCr, UP, IL-6, IL-12, MDA, T-SOD, p-Smad2/3, Smad7, TGF-β1, α-SMA, TLR-4, MyD88, p-JNK, p-ERK1/2, p-p38	MAPKs, TGF-β1/Smad	Diao et al. (2023)
	​	*In vivo*	5/6 nephrectomy (5/6Nx) -induced SD rats	10 mg/kg	8 weeks	Gavage	↑: Ccr, SOD, BMP-7, Smad 6 ↓: BUN, Scr, MDA, TGF-β	TGF-β/Smad	Zhang et al. (2019)
	​	*In vivo and* *In vitro*	NaAsO_2_-induced C57BL/6J mice HK-2 cell	10 mg/kg 1 μM	8 weeks 24 h	intraperitoneally —	↑: GPX4, GSH, SOD ↓: DUOX1, HIF-2α, IREB2, FPN, FTH, Iron, MDA, BUN, SCr, Ur, NGAL	HIF-2α/DUOX1/GPX4	Yang et al. (2024a)
​	​	*In vivo and* *In* *vitro*	UUO-induced male SD rats HK-2 cell	12.5 mg/kg 20 μM	2 weeks 24 h	Gavage —	↑: Par3 ↓: CTGF, PDGF-3, PDGFR-α, Caspase3, Caspase12, Egr-1, CHOP, GRP78	PDGF-C/PDGFR-α	Yao et al. (2022)
	DN	*In vivo and* *In vitro*	STZ-induced male ICR mice HK-2 cell	3 mg/kg 1, 5, 20 μM	18 weeks 48 h	Gavage —	↑: NF-κB, HO-1, NQO-1, GPx-1, p-AMPKα ↓: VCAM-1, glomerular PAS area, GSI, CoI, α-SMA, NOX-4, MCP-1, p65	Nrf2	Wu et al. (2016)
	​	*In vivo*	STZ-induced male SD rats	1 mg/kg	8 weeks	Gavage	↑: JG-12, TEER, claudin-5, VE-cadherin, CAT, SOD, LC3 II/I, bcl-1, Sirt1, Bnip3, FOXO3a, Atg5, Atg7, Atg12 ↓: TG, TCHO, HDL, LDL, GSP, BUN,SCr, 24-h urinary albumin, Ccr, VCAM-1, AGE, RAGE, RhoA, ROCK, p-MLC/t-MLC, Nox-4, MDA, 8-OHdG, ROS, NO, TNF-α, IL-6, IL-1β, ICAM-1, VCAM-1, p62	AGE-RAGE	Hou et al. (2017)
	SRNS	*In clinic and* *In vitro*	Conditionally immortalized human podocytes line	12.5, 25, 50 μM	24 h	—	↑: Nephrin ↓: SuPAR, uPAR	suPAR/uPAR-αvβ3	Li et al. (2021)
	SLE	*In vivo*	Pristane-induced female BALB/c mice	5 mg/kg	5 months	Gavage	↓: p-IKK, p-IκB, p-NF-κB	IKK/IκB/NF-κB	Lin et al. (2017)
SAB	AKI	*In vivo and* *In vitro*	Renal ischaemia-reperfusion (I/R) injury-induced male Balb/c mice HK-2 cell	50, 100, 200 mg/kg 1, 5, 10, 20, 40, 80 μM	7 days 24 h	Gavage —	↑: Nrf2, HO-1, SOD↓: SCr, BUN, C1-casp1/Pro-casp1, C1-IL-1β/Pro-IL-1β, C1-GSDMD/GSDMD, IL-1β, TNF-α, LDH, NLRP3, TXNIP, Histone H3, Keap1, MDA	Nrf2/NLRP3	Pang et al. (2020)
	​	*In vivo and* *In vitro*	Cisplatin-induced male C57BL/6 mice HK-2 cell	25, 50 mg/kg 0.25, 0.5, 1, 2, 4, 8, 16, 32, 64 μM	4 days 24 h	intraperitoneally —	↑: GPX4, AST/ALT, PRDX5 ↓: KIM-1, NGAL, ACSL4, COX2, SLC7A11, ROS, FSP1, MDA, GSH, SCr, BUN, IL-1β, TNF-α, MCP-1	PRDX5	Tao et al. (2025)
	​	*In vivo*	Renal ischaemia-reperfusion (I/R) injury-induced male SD rats	20, 40 mg/kg	7 days	intraperitoneally —	↑: SOD, CAT, GSH, PI3K, p-Akt/Akt ↓: SCr, BUN, MDA, MPO, NF-p65, IL-6, IL-1β, TNF-α	PI3K/Akt	Ma et al. (2017)
	CKD	*In vivo*	Sufentanil combined with dexmedetomidine-induced male SD rats	5, 10, 20 mg/kg	4 weeks	Gavage	↓: SCr, BUN, ALP, BMP-2, p-Smad 1, Smad 1, p-Smad 5, Smad 5, Osterix, Runx 2	BMP2/Smads	Ye et al. (2025)
​	​	*In vivo*	I/R/UUO-induced male C57BL/6J mice	40 mg/kg	10 days	Tail vein injection	↓: NEU1, ALK5, Smad2/3, TGF-β, KIM-1, Snai1/2, IL-6, IL-1β, TNF-α, CoIa1, CoI3a1, VIM	NEU1	Chen et al. (2023)
	​	*In vitro*	HK-2 cell	0.1, 1, 10, 100 mM	72 h	—	↓: α-SMA, E-cadherin, TGF-β	TGF-β	Pan et al. (2011)
	​	*In vitro*	HK-2 cell	0.1, 1, 10, 100 μM	72 h	—	↓: α-SMA, E-cadherin, TGF-β	TGF-β	Yao et al. (2009)
	​	*In vivo and* *In vitro*	Painlessly subjected to unilateral nephrectomy and injected with 5 mg/kg and 3 mg/kg (in saline) Adriamycin-induced male SD rats HK-2 cell	50, 100, 200 mg/kg 100 μM	6 weeks 24 h	Gavage —	↑: TP, ALB, Sirt1, Beclin1, LC3II/I ↓: CRE, TG, CHO, FN, α-SMA, TGF-β, p62	Sirt1-mediated autophagy	He et al. (2020)
	​	*In vivo and* *In vitro*	UUO/AAN-induced male C57 mice NRK-49F cells	10 mg/kg	13 days	intraperitoneally —	↑: PTEN ↓: SCr, BUN, FN, Fibronectin, α-SMA, p-Akt, EZH2, H3k27me3	PTEN/Akt	Lin et al. (2023)
	​	*In vitro*	HK-2 cell	1, 50 μM	48 h	—	↑: miR-106 b, miR-93, miR-25, E-cadherin, SDC1 ↓: α-SMA, TGF-β, HPSE	miR-106b-25	Tang et al. (2014)
	​	*In vivo and* *In vitro*	UUO-induced male C57BL/6 mice HK-2 cell	6.25, 12.5, 25 mg/kg 0.1, 1, 10 μM	2 weeks 24 h	intraperitoneally —	↑: E-cadherin ↓: BUN, SCr, FGF-2, α-SMA, TGF-β1	HPSE/SDC1	Hu et al. (2020)
	​	*In vitro*	The human ccRCC cell lines 786O and Caki-2	30 μM	24 h	—	↑: E-cadherin ↓: N-cadherin, YAP1	YAP1/Hippo	Chen et al. (2022c)
HChi-Ca-SAB	CKD	*In vivo*	UUO-induced male ICR mice	10 mg/kg	7 days	intravenously	↑: TP, ALB, Sirt1, Beclin1, LC3II/I ↓: FN, α-SMA, TGF-β, Hyp, MDA	TGF-β	Li et al. (2017)
	DN	*In vivo*	STZ-induced male SD rats	10 mg/kg	2 weeks	Gavage	↑: HDL, p-PI3K, p-Akt ↓: UTP, Urea, SCr, UCR, TG, IL-6, IL-1β, TNF-α, MCP-1, p-NF-κB	PI3K/Akt/NF-κB	Xu et al. (2024)
	​	*In vitro*	HMC cell	0.1, 1, 10 μM	24 h	—	↓: Fibronectin, p65-NF-κB, MMP-9, MMP-2	NF-κB	Luo et al. (2008)
​	​	*In vivo and* *In vitro*	HFD-induced male C57BL/6 mice HK-2 cell	3, 6.25, 12.5 mg/kg 1, 10, 100 μM	4 weeks 24 h	intraperitoneally —	↑: UO, Bcl2 ↓: URP, Cys-C, SCr, ICAM-1, IL-6, IL-1β, TNF-α, Bax, cleaved caspase-3, p-eIF2α, ATF4, CHOP, ATF6, IRE1α, XBP1s	Endoplasmic reticulum stress	Mai et al. (2020)
	​	*In vivo and* *In vitro*	STZ-induced male SD rats Rat mesangial cells (HBZY-1, iCell-r013)	50, 100, 200 mg/kg 0.1, 1, 10, 20, 40 μM	6 weeks 24 h	Gavage —	↑: SOD2, GSH-Px, SIRT3, LDH, Nu-FOXO1 ↓: SCr, MDA, FOXO1, CoIV, Fibronectin, ROS, IL-6, IL-1β, TNF-α, Ac-SOD2	SIRT3/FOXO1	Wen et al. (2023)
	​	*In vivo and* *In vitro*	Male diabetic db/db mice HK-2 cell	50 mg/kg 20 μM	6 weeks 24 h	Gavage —	↑: NLAP3, NF-κB, JC-1, HDL-C ↓: ADORA2B, NF-κB, ROS, TC, TG, LDL-C, ALT, AST, SCr, BUN, UA, IL-1β, TLR4	ADORA2B/NALP3	Wang et al. (2024)
	MN	*In vivo*	cBSA-induced male SD rats	100 mg/kg	2 weeks	Gavage	↑: miR-145-5p, Beclin1, LC3II/I ↓: SCr, BUN, IL-1β, IL-2, IL-6, TNF-α, CD68, PI3K, p-Akt	miR-145-5p/PI3K/Akt	Chen et al. (2022a)
	CIN	*In vitro*	HK-2 cell	10, 50, 100 μM	15 min	—	↑: SOD, CAT, GSH, PI3K, p-Akt/Akt ↓: p-IRE-1α, p-eIF-2α/eIF-2α, p-JNK, CHOP, Bax/Bcl-2, caspase-3, GRP 78, p-eIF 2 α, p-JNK, CHOP	Endoplasmic reticulum stress	Dong et al. (2021)
SAC	AKI	*In vivo*	Cisplatin-induced male ICR mice	5, 10, 20 mg/kg	10 days	intraperitoneally	↑: GSH, SOD, GPx3, Nrf2, HO-1, Sirt1, catalase, Bcl2 ↓: CRE, BUN, MDA, iNOS, COX-2, p-NF-κB, TLR4, TNF-α, IL-6, IL-1β, p-IKKα/β, p-IκBα, p-ERK, p-JNK, p-p38, Bax, c-caspase3, p-MAPK, p-CaMKK	CaMKK-AMPK-Sirt 1	Chien et al. (2021)
	CKD	*In vivo and* *In vitro*	UUO-induced male C57BL/6 mice NRK-49F cell	10 mg/kg 10, 30, 100 μM	2 weeks 48 h	intraperitoneally —	↑: Smad7 ↓: Fibronectin, SNAIL, N-Cadherin, Vimentin, p-Smad3	TGF-β/Smad-EMT	Wu et al. (2023)

Studies in different renal disease models indicate that the intervention effects of salvianolic acids are disease-specific. In acute kidney injury, all three compounds alleviate renal tubular epithelial cell damage by inhibiting novel forms of cell death such as pyroptosis and ferroptosis. For chronic kidney disease and renal fibrosis, SAB exhibits the most pronounced anti-fibrotic effects, involving multi-level regulation such as miRNA expression and epigenetic modifications. In diabetic nephropathy, both SAA and SAB ameliorate metabolic disorders and reduce damage from advanced glycation end products, though their emphases differ. Notably, the ability of SAA to improve podocyte steroid resistance in nephrotic syndrome, along with the inhibitory effect of SAB on mechanically driven tumor progression in renal cell carcinoma, expands the therapeutic scope of these compounds in kidney diseases.

Despite considerable progress in preclinical research, the clinical translation of salvianolic acids still faces multiple challenges. Pharmacokinetic studies have shown that all three salvianolic acids commonly suffer from low oral bioavailability, rapid *in vivo* metabolism, and insufficient tissue-specific distribution. Pharmacokinetic characteristics directly determine the *in vivo* efficacy of salvianolic acids: the short half-life and low bioavailability of SAB limit its long-term anti-fibrotic activity; the rapid clearance of SAA leads to insufficient renal exposure; the lowest bioavailability of SAC restricts its application in chronic kidney injury. These PK characteristics are consistent with their efficacy differences in CKD models. While formulation optimization strategies such as nanodelivery systems have partially addressed these issues, their application on a clinical scale remains limited. In terms of safety evaluation, existing data mainly stem from animal studies and small-scale human trials, with a lack of long-term, large-sample clinical safety evidence, particularly for SAC.

Future research should focus on establishing more robust pharmacokinetic-pharmacodynamic correlation models, developing kidney-targeted delivery systems, and validating efficacy and safety through well-designed clinical trials. Notably, the combination of salvianolic acids with the Traditional Chinese Medicine (TCM) *S. miltiorrhiza* or with existing Western drugs may yield synergistic effects. For example, the combination of SAA and metformin has shown enhanced efficacy in diabetic nephropathy, while SAB combined with tanshinone IIA synergistically regulates the PI3K/Akt/NF-κB pathway. These findings offer novel perspectives for integrated Chinese-Western medicine in the treatment of kidney diseases. Moreover, in-depth studies on the structure-activity relationships of salvianolic acids may guide the development of structural derivatives with improved activity and pharmacokinetic properties.

In summary, SAA, SAB, and SAC exert clear protective effects in various renal disease models by modulating multiple pathways, including oxidative stress, inflammation, cell death, and fibrosis. Although challenges such as bioavailability, targeted delivery, and long-term safety remain during clinical translation, advances in delivery technology, deeper mechanistic insights, and expanded clinical research hold promise for these natural compounds derived from TCM to provide new therapeutic strategies for the prevention and treatment of kidney diseases. Future studies should prioritize the development of precise delivery systems, confirmation of clinical efficacy, and exploration of synergistic effects with conventional therapies, thereby facilitating the substantial translation of salvianolic acids from experimental research to clinical application.

## 8 Conclusions and prospects

This article elucidates the mechanisms of action of SAA, SAB, and SAC in various kidney diseases, highlighting the significant protective effects of these natural compounds through multi-target synergistic regulation in areas such as antioxidant stress, anti-inflammation, anti-fibrosis, and regulation of cell death. The findings not only demonstrate the therapeutic efficacy of salvianolic acids in multiple renal disease models but also reveal their molecular-level interactions with key signaling pathways, including NF-κB, Nrf2, and TGF-β/Smad, providing a solid theoretical foundation for their clinical application. It is noteworthy that although the three compounds share overlapping core mechanisms, they still exhibit differences in specific target affinity, potency, and disease specificity. These distinctions offer important clues for the development of future precision-based treatment strategies. However, current research heavily relies on animal models, and high-quality clinical evidence remains limited. Additionally, all three salvianolic acids generally suffer from low oral bioavailability and rapid *in vivo* metabolism, and pharmacological and safety data on SAC in particular are still insufficient-all of which restrict their clinical translation. Therefore, there is an urgent need to improve their pharmacokinetic profiles through technological innovations such as novel nano-delivery systems, to conduct well-designed clinical trials to confirm their efficacy and safety, and to further explore potential synergistic effects when salvianolic acids are combined with TCM or Western drugs. Salvianolic acids hold promise for evolving from traditional herbal active ingredients into modern therapeutic options for kidney diseases, paving an innovative path for the integrated prevention and treatment of renal disorders through Chinese and Western medicine.
